# *Brukina* in Focus: A Narrative Review on Metagenomic Approaches to Fermentation and Food Safety

**DOI:** 10.1155/ijm/6677609

**Published:** 2026-04-28

**Authors:** Alexander Kwarteng, David Amedorme, Humphrey Precious Kwame Addy, Emmanuel Kobla Atsu Amewu, Priscilla Osei-Poku, Amma Larbi

**Affiliations:** ^1^ Department of Biochemistry and Biotechnology, College of Science, Kwame Nkrumah University of Science and Technology, Kumasi, Ghana, knust.edu.gh; ^2^ Kumasi Centre for Collaborative Research in Tropical Medicine, Kwame Nkrumah University of Science and Technology, Kumasi, Ghana, knust.edu.gh; ^3^ Animal Research Institute, Council for Scientific and Industrial Research, Accra, Ghana, csir.co.za; ^4^ Department of Biomedical Sciences, School of Allied Health Sciences, University of Cape Coast, Cape Coast, Ghana, ucc.edu.gh

**Keywords:** diversity, functional, sequencing, taxonomy

## Abstract

*Brukina*, a traditional fermented beverage smoothie made from milk and millet, is popular in Ghana and other West African countries due to its tasty flavor, high nutritional content, and affordability. Despite its widespread consumption, the nature of its production through artisanal fermentation processes presents concerns regarding microbial consistency, nutritional optimization, and food safety. This literature review explores the potential of metagenomic approaches to uncover microbial diversity, functional capacity, and safety profiles of *Brukina*. By integrating insights from amplicon‐targeted and shotgun whole‐genome sequencing studies on fermented foods, we highlight how next‐generation sequencing technologies can characterize lactic acid bacteria, yeast, and other microorganisms that drive fermentation. Additionally, we discuss how metagenomics can identify functional genes influencing carbohydrate metabolism, flavor and aroma generation, and production of antimicrobial resistance compounds. Thus, metagenomics provides a powerful framework for assessing public health risks and nutritional benefits. Bioinformatic tools have also been highlighted, and their relevant application in analyzing sequenced data to achieve taxonomic classification, identification of biochemical pathways, and functional profiling of microbial ecology of fermented foods. This review outlines key research gaps and recommends future directions, including starter culture development, standardization of *Brukina* production, multi‐omics integration in metagenomics, and microbiome‐informed food safety standards. Metagenomic profiling of *Brukina* holds promise for improving product quality, consumer safety, and scientific understanding of traditional fermented foods. By tackling the challenges raised, metagenomic techniques can be extremely helpful in maximizing *Brukina* fermentation, guaranteeing food safety, and maintaining the customs that give this product its distinctive character.

## 1. Introduction

A significant portion of many African nations′ diets and cultural traditions revolve around fermented foods. Among these, *Brukina*, a traditional fermented beverage made from milk and millet, is popular in Ghana and other West African countries due to its tasty flavor, high nutritional content, and affordability. Its original name is “Dègèr” in Burkina Faso, where it originates, but has been nicknamed *Brukina* in Ghana [1]. It is believed that because Ghana and Burkina Faso share a common border, the communities along the border tend to share culture and food. Hence, through this interchange of culture and food, “Dègèr” became a traditional drink for both countries. In Ghana, its production is dominated in the northern region, but in recent years, it has become popular in the southern belt and spread across the country [1, 2]. Among young people, particularly students and the general public, *Brukina* has grown in popularity and patronage as one of Ghana′s most well‐liked and native drinks. Due to its substantial clientele, *Brukina* effectively competes with other well‐known regional beverages like *nunu*, *asana*, *ice-kenkey*, and *sobolo* [3]. In Ghana, *Brukina* is typically packaged in plastic bottles, but it can also be served in wooden or plastic bowls by stationary vendors.

*Brukina* is predominantly a fermentation product, which accounts for its distinct flavor and richness. In recent years, it has been established that fermentation not only provides a way of preservation but also enhances the organoleptic characteristics and health advantages of milk [4]. Another category of probiotic‐rich functional meals is drinks made from cereals like millet. Cereals that have undergone fermentation have fewer total carbohydrates and more bioavailable forms of B vitamins and amino acids [5, 6]. According to a study by [7], foods made from fermented cereals provide health benefits because of their high nutritional content, which includes dietary fiber, protein, vitamins, and minerals. Additionally, the probiotic organisms found in fermented milk support urogenital health, immune system function, metabolism, and gastrointestinal tract balance [4, 8–10]. Probiotic carriers may be made possible by cereal ingredients such as soluble fiber, nondigestible carbohydrates, and different phytochemicals like phenolic compounds, antioxidants, and phytoestrogens. Therefore, given that *Brukina* is a combination of milk and cereal, it is believed to be a good source of probiotics and other vital nutrients to assist growth and development [1]. This is particularly important for a developing country like Ghana, where indigenous food products are essential sources of nutrients and are affordable for many families [3, 11].

*Brukina* is made by spontaneously fermenting milk or, in some cases, back‐slopping with previously fermented milk [1, 4]. However, because milk has a high nutrient content and an almost neutral pH, it is believed that leaving it at room temperature can foster the uncontrolled development of varied microbial populations [12]. Milk products are especially sensitive to temperature and other manufacturing conditions. As a result, it is critical to regulate milk operations throughout the value chain to ensure products are safe, nutritious, and suit customer sensory needs [8]. Since *Brukina* has been placed in the category of street foods, its production and vending are not properly supervised. However, one cannot discount the importance of street foods like *Brukina* in providing readily available (nutritious and convenient) food to thousands of customers daily [13, 14].

Several studies have sought to explore the microbiome of milk fermented products such as *Brukina* by deploying microbiological processes, but most are limited in scope because they rely on culture‐dependent approaches [15, 16]. However, due to the advances made in next‐generation sequencing (NGS) technologies, entire microbial communities of fermented foods can be studied comprehensively. This has produced the fast‐advancing field of metagenomics, where both culturable and nonculturable microbial ecologies can be explored. In this review, we explore the metagenomic approaches that can be leveraged to study fermented food, focusing on *Brukina*, a popular indigenous fermented milk product in Ghana.

## 2. Overview of *Brukina*

### 2.1. Traditional Preparation of *Brukina*

**Figure 1:**
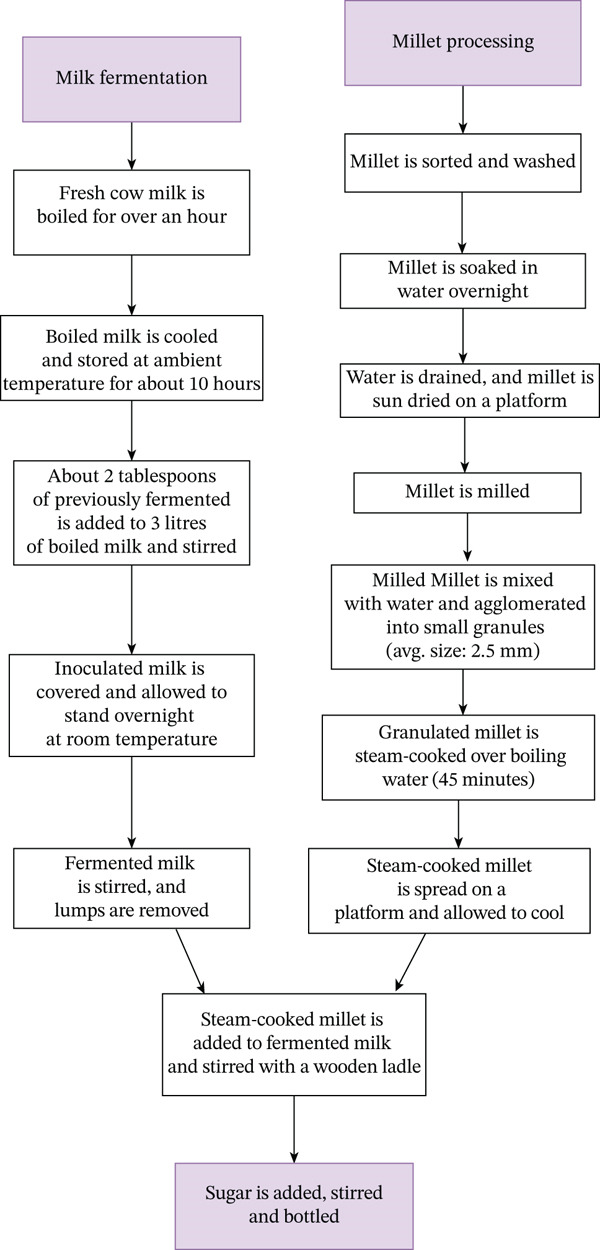
Flowchart of Brukina preparation.

*Brukina* is produced by blending fermented milk, sugar, and milled steamed millet. To begin, the millet is sifted to remove any potential contaminants from the original raw grain. It is then air‐dried, crushed in a mortar to remove the initial coat (epidermis), and ground into powder after three cleanings to eliminate any leftover material. After that, salt is dissolved in water, and the saltwater solution is sprinkled over the dough and well mixed. The resulting dough is steamed over hot boiling water using a steam cooking technique where the vapor from the boiling water is used to cook the millet dough until it forms a compact dough [2]. The crushed dough is allowed to cool before being manually mashed with a masher to produce smaller, coarser granular gravels. Margarine is rubbed into the coarse pebbles to promote uniform formation and flavor for the granular millet. Until used, it is stored in a container, covered, and kept at room temperature [1, 2]. Fresh milk fermentation for *Brukina* beverages is done traditionally using the producer′s indigenous expertise rather than according to any set standards. After being heated to a boil, raw milk is poured into a plastic basin. The milk is allowed to cool at room temperature after boiling. This is done for about 10 h. Starter cultures from previously fermented milk are added to the milk, which is then covered and allowed to ferment overnight. The final product, *Brukina*, is arrived at by mixing the fermented milk, steamed‐cooked millet, and sugar as a sweetener. *Brukina* is often served chilled in a bottle or any convenient container. Figure 1 is a *Brukina* preparation flowchart showing the step‐by‐step procedure for arriving at a final product of fermented milk and millet smoothie. This is adapted from [1] and edited.

### 2.2. Microbial Ecology of *Brukina*

The artisanal techniques of producing *Brukina*, coupled with environmental conditions that do not always adhere to strict hygienic practices, can result in significant variation in microbial content. Hence, the microbial content could vary significantly. The microbiota is largely influenced by exposure to the environment, storage conditions, and the microbiota of the raw ingredients. However, the rich microbial community that drives fermentation in *Brukina* is mostly made up of lactic acid bacteria (LAB) and ambient microorganisms. *Streptococcus*, *Lactobacillus*, *Leuconostoc*, and *Lactococcus* are frequently dominant bacterial genera [1, 3]. These bacteria greatly influence the texture, flavor, and control acidity of the *Brukina* drink while inhibiting the growth of spoilage organisms. In addition to the LAB, other microorganisms like yeasts, *Saccharomyces cerevisiae*, and *Candida* spp. may also be present, contributing to the mild production of alcohol, and also flavor and taste [16]. The presence of millet as a major raw material could activate the presence of fiber‐fermenting bacteria such as *Prevotella*, *Faecalibacterium*, and *Ruminococcus*, as seen in other cereal‐based fermented drinks and foods [17, 18]. Sometimes, the preparation process and vending conditions of *Brukina* expose the drink to contaminants that cause spoilage and other organisms that are carriers of disease‐causing pathogens, detrimental to the health of consumers [3]. Studies have shown the presence of pathogens such as E. coli O157:H7, *Staphylococcus aureus*, *Enterobacter*, *Yersinia*, *Sarcina*, and Aflatoxins [1, 3, 19–21].

### 2.3. Metagenomic Approaches in Fermented Foods

#### 2.3.1. Metagenomics

**Figure 2:**
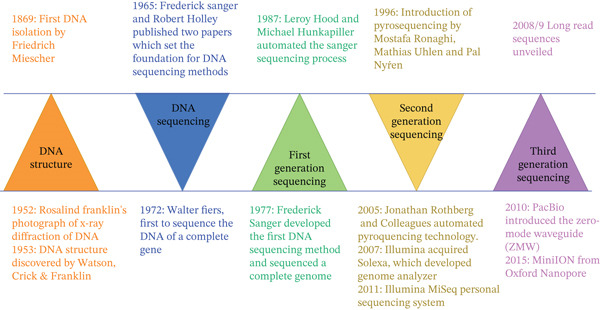
History of DNA sequencing.

The journey of DNA sequencing began with the discovery of DNA in 1869 and the unveiling of its double helix structure in the 1950s. The development of Sanger′s sequencing method in the 1970s and its subsequent automation in the 1980s laid the foundation for modern genomics. The rise of NGS in the early 2000s dramatically increased sequencing speed and accessibility (Figure 2) [22, 23]. NGS, also known as high‐throughput sequencing, has elevated traditional environmental (ecosystem) research to a new level and completely transformed the subject of microbiological ecology [24]. The field of “metagenomics,” which is the direct genetic analysis of an entire microbial community in environmental samples without the need to first develop clonal cultures, was created as a result of the use of this kind of cutting‐edge technology [25]. Metagenomics combines NGS and bioinformatic tools to study microbial ecology in its entirety for a given sample or environment. While some studies specifically define metagenomics as the untargeted shotgun sequencing of a community′s entire genomic content (DNA or RNA) [26, 27], others use metagenomics as a catch‐all term that includes long‐read, shotgun, or metabarcoding techniques (also known as amplicon metagenomics or metataxonomics) [28, 29]. In essence, metagenomics avoids the genetic variety and unculturability of the majority of microorganisms, which are the principal barriers to advancement in environmental and clinical microbiology [28]. Thus, when metagenomics is contextualized as a research technique, meta emphasizes the requirement of developing computational techniques that optimize knowledge of the genetic makeup and behaviors of communities [24]. Because of their complexity, these communities can only ever be sampled and never fully explained. But in the context of a field of study, “meta” describes the new science′s effort to understand biology at the level of the entire communities, focusing on the genes in the community rather than the individual organism [24,30], and, thus, examine how genes could affect one another′s actions to serve collective functions [30]. The term metagenomics was initially used to refer to sequence‐based functional analysis of collective microbial communities but has also been used for polymerase chain reaction techniques that target a specific gene of interest [31]. This gave rise to two main approaches, shotgun sequencing and targeted amplicon sequencing. Metagenomics not only provides bases for phylogeny analysis, as seen for instance in single‐gene targeted amplicon sequences such as 16S rRNA, but also access to functional gene composition, which is a step further in understanding microbial communities holistically [32, 33].

### 2.4. Microbial DNA Extraction

DNA extraction is crucial to the success of any metagenomics studies, particularly studies that rely on high‐throughput sequencing technologies. This is because DNA extraction precedes sequencing; as such, the quality of DNA extracted directly influences sequencing outcomes, though other factors can equally affect sequencing. An extracted DNA is the primary raw material for sequencing [26]. For instance, parameters of extracted DNA (concentration of the DNA and length of fragment) and instruments used also significantly affect the library preparation for the actual sequencing [34]. To overcome these difficulties, pre–DNA extraction processing techniques have been created for traditional fermented foods that are difficult to prepare, for example, viscous and sticky fermented meals [35–37]. There are several commercial and noncommercial DNA extraction kits available, and each approach has advantages and disadvantages based on the sample type and sequencing method employed [38]. Commercial kits for extracting DNA can be costly and might not work with very conventional fermentation systems. But one benefit of them is that they are standardized [35]. An additional consideration in assessing the quality of DNA collected for metagenomic sequencing is contamination from nonmicrobial or host DNA, usually human or animal [39]. For accurate sample analysis, cost, and time effectiveness, the removal of host DNA is required before the sequencing [40]. This process is very important to achieving a quality sequence dataset, which consequently imparts the reproducibility and reliability of the project being carried out.

### 2.5. Sequencing Platforms

DNA sequencing and its associated field of genomics are comparatively recent domains of study. After publishing techniques for RNA sequencing in the late 1960s, Dr. Frederick Sanger′s lab at the Medical Research Council (MRC) in Cambridge, United Kingdom, started working on developing a DNA sequencing technique in the early 1970s. This eventually led to the development of Sanger sequencing as one of the earliest sequencing technologies that significantly aided in the creation of automated DNA sequencers [41]. Sanger sequencing has been a pacesetter, and it is often referred to as the gold standard for sequencing [24]. Sanger sequencing is also known as the chain termination method and uses dideoxynucleotides (ddNTPs) to terminate DNA replication to determine the sequence of DNA [41]. Despite being comparatively slow by today′s NGS standards, advancements in automation, commercialization, and the chain termination methodology make it a reliable technology for many applications. Sanger sequencing involves three main processes. The first process is a polymerase chain reaction (PCR), which follows the conventional PCR format and additional modification involving dideoxynucleotides (ddNTPs). The second step is a size separation of the chain‐terminated oligonucleotides using gel electrophoresis. The third and last step is an analysis of the gel electrophoresis and determination of the DNA sequence [41, 42].

The low throughput and high cost associated with Sanger sequencing led to the emergence of a category of sequencing technologies referred to as second‐generation sequencing (SGS). The launch of Roche 454′s pyrosequencing system in 2005 marked the beginning of the massively parallel sequencing revolution. Next came Illumina/Solexa′s sequencing‐by‐synthesis‐based Genome Analyzer platform in 2007 and ABI′s sequencing‐by‐ligation SOLiD system [43]. The SGS dominated for a while, and then, due to the demand for speed and larger read size, the third‐generation sequencing technologies were developed. The third‐generation sequencing technologies worked more effectively by directly targeting single DNA molecules, thus enabling real‐time sequencing. Three significant enhancements have been made to TGS platforms: Read lengths will be increased from tens of bases to tens of thousands of bases per read; sequencing times will be shortened from days to hours (or minutes for real‐time applications); and sequencing biases brought about by PCR amplification will be reduced or eliminated [44, 45].

Sequencing DNA is the bedrock of metagenomics. Sanger sequencing has evolved over the years, giving rise to other technologies that have dramatically improved the robustness of sequencing and reduced the cost [24]. Many of the current metagenomic and short‐read applications were started by Next‐Generation Sequencers, which were made possible by major advancements in sequencing technology. The Illumina suite of sequencers, which was invented by Roche 454, Illumina, and Ion Torrent, is used in the majority of metagenomic research [24, 46]. The sequencing by the synthesis method used by Illumina platforms employs fluorescently labeled nucleotides, which are integrated by DNA polymerases that are complementary to the template DNA strand on flow cells. When an instrument is incorporated, a camera within it takes pictures and emits light with a particular wavelength. After that, the pictures are converted into DNA sequences, one base at a time [47, 48]. In response to the increasing need for improved sequencing methods, recent Illumina releases are focused on increasing throughput capacity and cost‐effectiveness while reducing error rates. One of Illumina′s latest products, the NovaSeq 6000, allows for industrial‐scale sequencing and, in comparison to earlier versions, generates up to 6 Tb and 20 billion reads at the lowest cost per base. Error rates have also dropped with the NovaSeq 6000 and HighSeq X Ten, the latter of which is the least expensive human genome sequencing [49]. Other platforms available on the market include Oxford Nanopore Technology (ONT) and PacBio, which are primarily long‐read technologies that have been used in shotgun whole‐genome sequencing (WGS) as well as targeted amplicon sequencing [48, 50]. A summary of DNA sequencing equipment, protocols, and applications is presented in Table 1.

**Table 1:** Summary DNA sequencing equipment, protocols, and applications.

Sequencer	Protocol/kit	Type	Use case
Illumina MiSeq	MiSeq Reagent Kit V2/V3, Nextera XT, Truseq Amplicon	Short read	Targeted amplicon/small WGS
Illumina NextSeq	Nextera DNA Flex (now Illumina DNA Prep)	Short read, midthroughput	Shotgun WGS/targeted amplicon
Illumina NovaSeq	TruSeq DNA Nano/DNA PCR‐Free	Short read, ultra‐high throughput	Shotgun WGS
Oxford Nanopore MinION	Ligation Sequencing Kit/Rapid Barcoding Kit	Long read	Shotgun WGS/targeted amplicon
Oxford Nanopore GridION	Ligation Sequencing Kit/Rapid Barcoding Kit	Long read	Shotgun WGS/targeted amplicon
PacBio Sequel IIe	SMRTbell Express Template Prep Kit 2.0	Long read, HiFi	Shotgun WGS/targeted (HiFi amplicons)
Thermo Ion Torrent S5	Ion AmpliSeq Library Kit Plus	Short read	Targeted amplicon sequencing
Thermo Ion Proton	Ion Xpress Plus Fragment Library Kit	Short read	Shotgun WGS/targeted amplicon
Qiagen GeneReader	QIAseq Targeted DNA Panel	Short read	Targeted amplicon sequencing
MGI DNBSEQ‐G400	MGIEasy FS DNA Library Prep Kit	Short read	Shotgun WGS/targeted amplicon

### 2.6. Library Preparation Methods

From sample preparation to final results analysis, sequence analysis procedures, like any other laboratory technique, require guidelines and protocols that detail the process and ensure consistent execution of the work. Library preparation is an essential step for quality and a successful sequencing procedure. It is a crucial process carried out during sequencing [51]. There are many different approaches for preparing NGS libraries depending on the sequencing platforms being used. However, they all include fusing (fragments of) DNA or RNA molecules with adapters that have the components required for solid surface immobilization and sequencing. A review of this process over time shows that it is cumbersome and costly, as it involves several steps and expensive reagents [51, 52]. The cost involved in library preparation is subjective and varies from laboratory to laboratory. One main solution being applied is automation. Automation not only reduces the cost buildup but also enables the reproducibility of the procedure. The automation process has been applied to the different available sequencing platforms.

The procedures and automation requirements of many well‐established methods for various sequencing platforms are identical [48, 51]. There are five possible automation procedures to consider: the process of mechanical fragmentation, reactions with enzymes, selecting sizes and cleaning up, amplification, and measurement.

### 2.7. Sequencing Approaches

#### 2.7.1. WGS

WGS is an approach to sequencing DNA whereby the entire genome of the organism is targeted for sequencing. It involves sequencing both coding and noncoding regions of the genome [34]. With the advances in sequencing platforms and technology, WGS has seen a significant boost, particularly in the decline in cost, faster sequencing, and accuracy. WGS has increased in scope, and it has been applied to many more organisms, from plant species to mammals and microorganisms [53–58]. Some notable advances that have improved WGS include long‐read sequencing. For instance, HiFi circular consensus sequencing offers a contig N50 of >15 megabases (Mb) and accuracy of 99.9%, making it ideal for de novo genome assembly and structural variant detection [59]. Also, Oxford Nanopore Technologies, which offered ultra‐long reads for WGS, is good at identifying identical repeats, making it suitable for resolving complex genomic regions [60]. In general, WGS makes it possible to identify genetic differences, from single‐nucleotide polymorphisms (SNPs) to more significant structural alterations like insertions, deletions, and rearrangements, by sequencing every DNA molecule in an organism′s genome [48, 61]. Many methods and tools have been developed to analyze WGS data. Bioinformatic software such as HiCanu and hifiasm are used for genome assembly. Variant detection tools like Genome Analysis Toolkit (GATK) and SAMTools allow for the accurate detection of genetic variants, including SNPs and structural variants [62–64].

#### 2.7.2. Shotgun Sequencing

In microbial ecology, metagenomics, the shotgun method is frequently employed to sequence the complete population of microorganisms in a particular habitat [65, 66]. The metagenomic shotgun technique, when used on mixed microbial DNA from an environment, resembles the objective of WGS. The whole genome is targeted in this case, rather than just particular areas of the organism′s genomic locus [67]. Shotgun is a technique that first randomly fragments the targeted genome before sequencing each fragment separately [68]. The fragments are later joined together using computer algorithms to assemble the overlapping sequences into a continuous sequence [67, 68]. As a result, some of these readings will come from genomic loci that are taxonomically informative (like 16S), whereas others will come from coding sequences that reveal information about the biological functions that the genome encodes. Shotgun sequencing for metagenomics, therefore, offers the chance to investigate two facets of a microbial community at the same time: specific microbial presence and corresponding genomic function [69].

#### 2.7.3. Targeted Amplicon Sequencing

In amplicon sequencing, highly conserved bacterial genes are amplified, and their regions sequenced [70]. The sequences are then compared to an existing database to identify the bacterial organisms from which they originated. These regions are sometimes referred to as marker gene sequences, such as the intergenic transcribed spacer (ITS) and 16S/18S/26S rRNA [66, 71]. They have the following qualities listed: it exists in all members of a community, varies solely and consistently between people with different genomes, and varies in proportion to the evolutionary distance between individuals with different genomes [35, 72]. Being present in all bacterial cells, the 16S ribosomal RNA subunit gene (16S rRNA) is a universal target since it is necessary for the initiation of protein synthesis and mRNA translation. It is one of the most frequently used markers and has been chosen for metabarcoding in bacterial genomes because it is highly conserved across almost all bacterial species, allowing the use of universal primers, and because hypervariable areas allow for bacterial identification and taxonomic classification [73]. Short‐read sequencing is only capable of sequencing a subset of the 16S rRNA gene′s hypervariable regions (designated V1 through V9). Amplicons up to 450 bp in length, such as V1–V3 or V3–V4 sections, are commonly targeted by PCR for sequencing. The type of sample source determines how appropriate the hypervariable regions are. There is still disagreement in this field since the hypervariable areas that are targeted by various studies and for particular bacterial genera vary [26, 74]. Regardless of the issue with the selection of hypervariable regions employed, 16S rRNA sequencing has been widely used in the metagenomics field, especially for the V3–V4 area in conjunction with Illumina sequencing [75, 76]. The 16S rRNA gene is easy to sequence and reasonably priced, in addition to fulfilling the previously mentioned requirements for an “ideal” marker [72, 77].

### 2.8. Microbiome Bioinformatic Pipelines

#### 2.8.1. Shotgun Metagenomics Pipelines

A shotgun metagenomics pipeline is a collection of bioinformatics tools and methods used to analyze metagenomic data generated from the shotgun sequencing approach [78]. This type of metagenomic data is usually all the available DNA sequences from a mixed microbial community. The pipeline aims to characterize the diversity, abundance, and potential function of the characterized microorganisms present in the given sample. Many pipelines exist for shotgun sequence analysis. Some include Just A Microbiology System (JAMS) [79], Whole MetaGenome Sequence Assembly pipeline (WGSA2) [80], Sunbeam [81], and MEDUSA [82]. Kraken2, a k‐mer‐based classifier, is used in both WGSA2 and JAMS [82]. A marker gene technique created by the Huttenhower Lab is an additional method for profiling shotgun metagenomics sequencing. Known as Metagenomic Phylogenetic Analysis (MetaPhlAn), the marker gene technique can be applied within the bioBakery range of bioinformatics tools. MetaPhlAn is available in three versions as of 2023: version two (MetaPhlAn2) and version three (MetaPhlAn3) are based on marker genes, while version four (MetaPhlAn4) is based on both marker genes and MAG.

The key steps in a typical shotgun metagenomics pipeline include quality control, genome assembly, binning of contigs, taxonomy assignment, and functional annotation. There are several bioinformatic tools available for each step; as a result, researchers are faced with the daunting task of choosing bioinformatic tools that suit their competence, available computational power, and resources at their disposal. More importantly, the aim of the study should be the driving factor in selecting the best tools for each task. It is also important to settle on tools commonly used and recommended by the community of scientists in the field. A summary of bioinformatic tools that are available for use for each stage of the shotgun sequence data analysis is presented in Table 2.

**Table 2:** Summary of bioinformatic tools for shotgun sequence analysis.

Step	Application	Common tools	Notes	Reference
Quality control	Assess and clean raw reads	FastQC, MultiQC, fastq, Trimmomatic	FastQC for assessment, MultiQC aggregates FastQC results fastp/trimmomatic for filtering and trimming,	(Bhanu et al., 2020; Bolger et al., 2014; Chen et al., 2018; Ewels et al., 2016)
Host/contaminant removal	Remove non‐target DNA	Bowtie2, BWA, BBMap	Align reads to host genome, keep unmapped reads	(Langmead & Salzberg, 2012)
Assembly	Assemble reads	MEGAHIT, SPAdes, IDBA‐UD	MEGAHIT is specially noted for ultra‐fast genome assembly; SPAdes is for de novo genome assembly	(Li et al., 2015; Peng et al., 2012; Prjibelski et al., 2020)
Gene prediction	Identify coding regions	Prodigal, MetaGeneMark	Gene prediction	(Gemayel et al., 2022; Larralde, 2022)
Taxonomic classification	Assign taxonomy	Kraken2, MetaPHLAn, Centrifuge, Kaiju	Taxonomy profiling	(Blanco‐Míguez et al., 2023; Wood et al., 2019)
Functional annotation	Group contigs into draft genomes (MAGs)	MetaBAT2, MaxBin2, CONCOCT	Metagenomic contig binning	(Alneberg et al., 2014; Kang et al., 2019; Xue et al., 2019)
MAG quality assessment	Evaluate completeness/contamination	CheckM2, QUAST, BUSCO	Quality assessment tool for evaluating and comparing genomes	(Chklovski et al., 2023; Gurevich et al., 2013; Seppey et al., 2019)
Visualization	Create figures and summary tables	GraPhlAn, Phyloseq, Anvi′o	Krona for interactive taxonomy plots, Anvi′o for MAGS	(Eren et al., 2015; Huttenhower, 2016; McMurdie & Holmes, 2013)
Statistical analysis	Compare communities/metadata	QIIME2, DeSeq, STAMP	Used for alpha/beta diversity, differential analysis	(Bolyen et al., 2019; Love et al., 2014; Parks et al., 2014)

#### 2.8.2. 16S Microbiome Pipelines

The 16S rRNA microbiome of a given sample is explored using a set of procedures that are often referred to as bioinformatic pipelines or workflows. These workflows help to analyze 16S rRNA datasets to identify and classify bacteria populations within the sample. The pipelines involve several steps, including data processing, quality control, taxonomic analysis, and functional analysis using tools such as QIIME, Mothur, and USEARCH [83]. DADA2 [84], QIIME 2 [85], and Mothur [86] are now the most widely used software programs for analyzing the 16S amplicon sequencing data. Some of these software packages have undergone consistent updates. Both QIIME 2 and Mothur were created following the invention of NGS technologies. They both essentially use a similar workflow, which involves reads being grouped based on sequence similarity into operational taxonomic units (OTUs) or denoised OTUs (often referred to as amplicon sequence variants, or ASVs), depending on whether full sequence identity is required for clustering. To increase computing efficiency by reducing the number of sequences that must be aligned to a large number of reference genomes and to account for the modest levels of genetic variation seen within a particular bacterial strain, the first clustering phase helps to mitigate sequencing errors. As such, consistently, these packages′ developments move along with developments in NGS technologies [47]. For instance, QIIME2 has released an updated version of its package, which is a distribution focused on targeted amplicon sequences known as the QIIME2 Amplicon Distribution package with several plugins for enhanced analysis. OTU classifications were initially based on a 97% similarity index, but as updates continue to unfold, new classifications are now made on 99% to 100% similarity indexes [87, 88]. As an alternative to OTU clustering, PathoScope 2 aligns reads directly against a reference genome library. PathoScope dampens any sequencing mistakes and slight genetic variation by reassigning ambiguously matched reads using a Bayesian mixed modeling approach [89]. Another alternative is Kraken2. Kraken 2 looks for k‐mers without alignment against a reference genome library and assigns taxonomic classifications to each read by counting the number of k‐mer matches in a complete read against every taxonomic node in its reference library [84]. These features in Kraken 2 and PathoScope have been able to resolve the inherent challenges found in OTU generation.

#### 2.8.3. DADA2 Pipeline

Divisive Amplicon Denoising Algorithm 2 (DADA2) is an error correction methodology commonly known as denoising, which provides high resolution for specific species and strain level classification [84, 90]. It is used in microbiome analysis to process raw read sequences and infer exact ASVs by removing errors while preserving biological variation [84]. This approach was mainly developed to resolve the issues that arise when taxonomic classification is done using the clustering OTU method, particularly in Illumina‐sequence amplicon data [84]. Without coarse‐graining into OTUs, DADA2 infers sample sequences precisely and fixes variations as small as one nucleotide. The complete sequencing run is used to train a parametric error model that DADA2 creates, which is then used to correct and collapse the sequence errors into what are known as amplicon sequence variations (ASVs) [91]. The key features of DADA2 include error‐based model correction. This feature appears to be the fundamental algorithm of DADA2. Another feature is quality filtering of sequence reads. The quality filtering involves trimming of low‐quality reads, thus ensuring high‐confidence ASV identification and removal of chimera sequences (artifacts of PCR amplification). DADA2 also has a seamless integration model that works well with databases such as SILVA and GreenGenes [84]. These key features are integral to the workflow of DADA2. The pipeline steps include filtering bases in the sequence reads that are ambiguous, followed by identifying and removing primers. The quality profile of the forward and reverse reads is plotted and saved as a quality profile in a PDF file. The next main step in the pipeline (workflow) is dereplicating sequence reads using derepFastq. For paired‐end sequence reads, the reads are merged using mergePairs. After all these processes, the ASVs are classified using the assignTaxonomy algorithm plugin [84].

#### 2.8.4. QIIME2 Pipeline

Quantitative Insights into Microbial Ecology 2 (QIIME 2) is a next‐generation bioinformatics software package (pipeline) designed for the analysis of amplicon sequencing data, including 16S rRNA, Internal Transcribed Spacer (ITS), 18S rRNA, and other marker gene studies. With an emphasis on data and analysis transparency, QIIME 2 is a robust, extendable, and decentralized microbiome analysis software [85]. This version of QIIME is a complete overhaul of QIIME 1, the original version of the QIIME bioinformatics tool. With QIIME 2, researchers can begin an investigation with raw DNA sequence data using QIIME 2 and end up with statistical results and figures suitable for publication. Important features include automated and integrated data provenance tracking, a system of semantic types, and a system of plugins that increase the functionality of microbiome analysis and support for a variety of user interface formats, including graphical, command line, and application programming interface (API) [85, 92, 93]. In terms of specific features and their functions, some notable examples include a plugin‐based architecture that allows users to customize their workflows depending on the type of analysis they want to carry out. Another feature is denoising and ASV inference. This feature allows users to use DADA2 or Deblur to infer ASVs, which are more accurate in taxonomic classification than OTUs [94]. The taxa plugin is used for feature taxonomy annotation by first collapsing features by their taxonomy at each specified taxonomy level and followed by filtering sequences and creating taxonomy‐based filter tables. The phylogeny analysis feature also allows for constructing phylogenetic trees for evolutionary relationship studies. With the diversity analysis feature, users can compute alpha diversity parameters (Shannon, Simpson, and Faith′s PD) and beta diversity parameters (Bray–Curtis, UniFrac, Jaccard, and others). Another important feature in QIIME 2 is data visualization, which produces interactive outputs using the qiime view plugin. These features and many more have drastically improved microbiome analysis and visualization. In the recent update, QIIME has released four distributions, which are the amplicon distribution, metagenome, pathogenome, and tiny [85, 93].

#### 2.8.5. PICRUSt2 Pipeline

Phylogenetic Investigating of Communities by Reconstructing of Unobserved States 2 (PICRUSt2) is the newest version of PICRUSt, designed to predict functional gene content from marker gene sequences such as 16S rRNA, 18S rRNA, and ITS. The PICRUSt2 is an improvement of the overall functionality of PICRUSt. Unlike PICRUSt, which operates by using closed‐reference OTUs, PICRUSt2 uses ASVs, which are generated by pipelines such as DADA2 or Deblur [95]. With its improvement, PICRUSt2 has features such as phylogenetic placement and expanded functional prediction, where PICRUSt2 conducts gene prediction beyond KEGG Orthology to include enzyme commission numbers, enzyme pathways, cluster of orthologous groups, and MetaCyc pathways [95]. PICRUSt2 also reduces biases in functional prediction by incorporating genomes from other databases, other than relying solely on the Greengenes database, as was the case in PICRUSt [96]. Application of PICRUSt2 has been carried out in different fields of microbiome analysis. In human gut microbiome analysis, PICRUSt2 has been deployed to predict functional potential in gut microbiomes, identifying pathways associated with health and disease states [97–99]. In soil and plant microbiome studies, PICRUSt2 has been employed to infer microbial functions related to plant–microbe interaction and biogeochemical cycling [100–102]. PICRUSt2 has also been applied in the studies of food microbiome, particularly fermented foods such as dairy products and fermented beverages, to predict pathways associated with lactic acid metabolism and quorum sensing [103–105].

### 2.9. Taxonomic classification

The taxonomic classification of metagenomic sequences is mostly done to catalog or categorize various microbial communities living in an environment, to identify the exact species of origin [106]. Because of this, taxonomic classification is a crucial step in many metagenomic applications, including tracking epidemics, analyzing microbiomes, and diagnosing illnesses. Oligonucleotide frequency is one of the most commonly generated sequence composition features utilized by contemporary taxonomy classification methods [107, 108]. There are various approaches available for the taxonomic classification of sequences. The RDP classifier is a naïve Bayesian classification approach that is widely used [109]. It makes use of a feature space made up of any conceivable eight‐length RNA sequence. The probability that the query sequence is part of a group of reference sequences from a certain taxonomic clade is determined by employing terms that appear in the query sequence, but whose taxonomic origin is unclear. Within each taxonomic rank, the query is assigned to the clade with the highest likelihood score [109, 110]. These taxonomic classifications are utilized mainly to evaluate the community structure, evenness, and diversity of microorganisms. Rarefaction curves can be used to determine a sample′s completeness as well as the species richness of a microbial community.

### 2.10. OTU Versus ASVs

In taxonomic classification, several methods are used to classify bacteria populations using criteria such as the similarity index or exact matching of sequences for comparison and identification [110]. In these approaches, the similarity index is adopted when the OTU method is being used, and identification of unique sequences at the nucleotide level is used when the ASV method is being used [111]. OTU generation involves clustering of similar species into one group, which usually results in the individual identity of the species being lost to the clustering group identity. As an alternative, some have attempted to reduce the risk of losing diversity to clustering by requiring extremely high levels of sequence identity; thresholds near 100% are used in this approach. However, there is a considerable risk of identifying sequencing errors as new species and false diversity [112]. The simplest method of conducting OTU clustering is by the method known as de novo clustering. This method, although it requires huge computational power, does not require the use of a referencing database but rather creates the clusters based on individual sequences [88]. Furthermore, if data are added to or deleted from the work, de novo clustering needs to be done again. This is due to the possibility that a single sequence may cluster differently based on which other sequences were found throughout the study. Another approach to OTU clustering is the reference‐based clustering method [88, 94, 113]. This method seems more computationally efficient than the non–reference‐based method. As the name suggests, this approach compares newly discovered sequences to a reference library of target gene sequences from recognized taxa. Because it is unlikely that a few incorrect SNVs will alter the final consensus sequence from the complete OTU, this approach will also reduce the impact of sequencing errors [94, 113, 114]. Furthermore, closed‐reference clustering will exclude the sequencing read from additional analysis if there are enough faults in the read to preclude it from grouping with a reference sequence. This method also allows for newly discovered taxa to be easily incorporated [113]. The only drawback with this method is that it relies solely on a reference database.

The ASV strategy aims to go opposite direction from OTU clustering approaches, which try to blend comparable sequences into an abstracted consensus sequence to reduce the impact of any sequencing errors within the read pool [115]. Finding out which specific sequences were read and how many times each was read will be the first step in the ASV process. After that, the read data are combined with an error model for the sequencing run, which allows for the possibility to compare similar reads and calculate the likelihood that a particular read at a particular frequency is not the result of sequencer error [94]. For every exact sequence, this essentially generates a *p* value, where the null hypothesis is equivalent to that precise sequence being the result of a sequencing mistake. This is subsequently filtered using a threshold value for confidence, and based on that, a group of exact sequences end up having the same confidence [116, 117]. Because an ASV has an exact sequence, it may be compared to a reference database at a much greater resolution, enabling more accurate identification down to the species level and possibly beyond. Additionally, a particular target gene sequence should always produce the same ASV [115, 117]. Numerous considerations support the idea that the field ought to adopt an ASV methodology. ASV techniques can offer a major benefit to more accurate microorganism identification, as previously mentioned. They can also give a more thorough image of the diversity in a sample, but in some cases, the results they generate are comparable [94, 116].

### 2.11. Taxonomy Classification Databases

#### 2.11.1. Silva Database

The SILVA (Structured and Improved Large‐Scale Alignment) database is one of the most widely used curated repositories for ribosomal RNA (rRNA) sequences, facilitating taxonomic classification and phylogenetic studies in microbiome research. This database was developed and maintained by the Max Planck Institute of Marine Microbiology and provides reference sequences for 16S, 18S, and 23S rRNA genes [118]. The specific features of the SILVA database include a comprehensive rRNA sequence coverage of bacteria, archaea, and eukaryotes. It also aligns with other public databases like NCBI taxonomy and Greengenes when computing taxonomic classification and integrates well with other bioinformatic tools like QIIME2 and PICRUSt2 [85, 95]. The classification that is generated using the SILVA database is ordered hierarchically. The SILVA database has been applied in the study of microbial community profiling. It has been used to accurately profile microbial communities in soil samples, gut microbiome, marine microbiome, and fermentation microbiome [3, 119, 120]. It has been applied in pathogen identification and microbial dysbiosis studies. Categorizing the microbial populations in the human respiratory tract, oral cavity, and gut has shed light on antibiotic resistance and infectious illnesses.

#### 2.11.2. Greengene Database

The Greengene database is a widely used database for microbial taxonomy classification and annotation for 16S rRNA gene sequences [121]. It was developed in the early 2000s and was essential to the taxonomy assignment of sequences from bacteria and archaea that were obtained from environmental samples. Greengenes was first published by [122] as a curated, chimera‐checked database that offered a standardized framework for phylogenetic research and microbiological taxonomy classification based on de novo tree inference [121]. As NGS technology gained popularity, Greengenes had a significant impact. It served as the foundation for widely used analysis tools like QIIME [93]. For a while, the Greengene database has not been updated, resulting in it falling behind other databases like Silva as a preferred option by many scientists in the community of metagenomics. However, there have been recent updates to the Greengene database with a new version known as Greengene2 [123]. Its phylogenetically consistent taxonomy, which was built from an extensive 16S rRNA gene tree and compatible with the NCBI taxonomy, was one of its main advantages. This facilitated the creation of bioinformatics pipelines for microbial community profiling and allowed for precise comparisons between investigations [123].

#### 2.11.3. Functional Classification

Functional classification methods aim to classify microbial communities based on their potential biological roles by inferring metabolic pathways, enzyme activities, and gene functions. Some commonly used databases in this process are the Kyoto Encyclopedia of Genes and Genomes, Enzyme Explorer, and MetaCyc [96, 102]. Some notable functional prediction tools used in microbial community studies are PICRUSt2 (Phylogenetic Investigation of Communities by Reconstruction of Unobserved States 2), HUMAnN3 (HMP Unified Metabolic Analysis Network), and FAPROTAX (Functional Annotation of Prokaryotic Taxa). In cases where de novo gene discovery techniques are deployed, the majority of gene detection and annotation is done via BLAST‐ or HMMER‐based homology searches [124]. This is because de novo gene discovery techniques typically do not offer a functional annotation directly. The unannotated sequence is then compared to a database of sequences with known functions to perform homology‐based functional annotation. When a sequence with an unknown function exhibits a high degree of resemblance to a database sequence with a known purpose, it is assumed that the two sequences have the same or extremely similar functions [125].

#### 2.11.4. Application to Fermented Foods—*Brukina*

Fermented foods like *Brukina*, a traditional West African milk‐cereal beverage, harbor complex microbial ecosystems that contribute to their unique flavors, textures, and health benefits [126]. Metagenomics offers a powerful approach to decode these microbial communities without the limitations of traditional culture‐based methods. By analyzing the collective genetic material present in these foods, researchers can gain unprecedented insights into microbial diversity, functional capabilities, and safety profiles [127, 128]. The application of metagenomics to Brukina and similar fermented products allows for a comprehensive characterization of both culturable and unculturable microorganisms, providing a deeper understanding of the entire microbial community. It enables the detection of rare but potentially important microbial species that might otherwise be overlooked. Additionally, metagenomics helps unravel the complex microbial interactions that occur during fermentation, shedding light on how these communities develop and function. This approach also facilitates the identification of beneficial compounds produced throughout the fermentation process, which can enhance the nutritional and health value of the food. Importantly, metagenomics aids in assessing potential food safety risks by detecting harmful microbes or antibiotic resistance genes, thereby supporting the development of safer and higher quality fermented products [127, 129].

#### 2.11.5. Functional Potential of the *Brukina* Microbiome

The microorganisms in the *Brukina* microbiome may not only influence fermentation but also contribute important functional benefits. Carbohydrate metabolism is often carried out by LAB. These microbes break down complex sugars into lactic acid, which enhances digestibility and aids in preservation [130, 131]. In terms of organic acid production, lactic and acetic acids lower the pH and prevent the growth of pathogens in fermented foods [132, 133]. Some strains of LAB also function in vitamin biosynthesis to produce folate, B2, and B12, and their presence could enhance the nutritional potential of *Brukina* [134]. By preserving a healthy microbiome, LAB strains enhance nutrient content and function as probiotics, enhancing gut health. *Lactobacillus acidophilus* and *Lactiplantibacillus plantarum* strains aid in immune system support and gastrointestinal problem prevention [135]. For aroma and flavor production, yeast and bacteria contribute to ester, aldehyde, and alcohol production [136]. Esters, aldehydes, and higher alcohols are among the metabolites that add to its distinct flavor profile and improve the finished product′s overall sensory qualities [137]. Functional prediction methods such as HUMAnN3 or PICRUSt2 can be used to infer these functions, and shotgun metagenomics can be used to confirm them. Identification of probiotic strains and fermentation optimization depend on such findings.

#### 2.11.6. *Brukina* Microbial Genomics Potential Workflow

**Figure 3:**
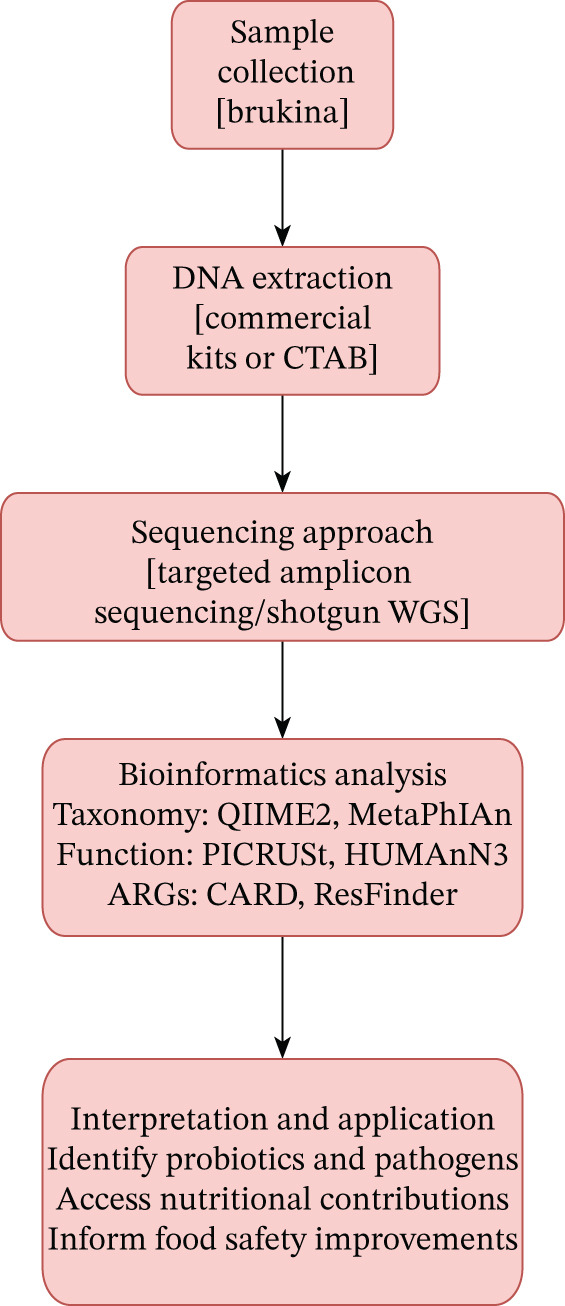
*Brukina* metagenomics workflow.

Metagenomics offers a powerful and comprehensive method for studying the microbial communities present in fermented foods such as *Brukina*. To apply this approach, high‐quality DNA from *Brukina* samples would need to be extracted using either commercial extraction kits or the CTAB method. Sequencing using targeted amplicon sequencing (e.g., 16S rRNA gene) or shotgun WGS, depending on the level of detail required, would be performed, after which the sequencing data will be analyzed with bioinformatics tools like QIIME2 and MetaPhlAn for taxonomic profiling, PICRUSt and HUMAnN3 for predicting microbial functions, and CARD or ResFinder databases to identify antibiotic resistance genes. By interpreting these results, researchers can pinpoint beneficial probiotic strains and potential pathogens, evaluate the nutritional impact of the microbial community, and develop informed strategies to enhance the safety, quality, and health benefits of fermented foods such as *Brukina*. The diagrammatic representation of such a potential workflow is shown in Figure 3.

### 2.12. Limitations and Challenges

#### 2.12.1. Sample Complexity and Representativeness

The complex nature of fermented foods, such as milk‐based fermented foods like *Brukina*, could hinder the study of fermentation microbiomes. At the onset, challenges of sample collection and handling, quality DNA extraction and quantification due to the presence of lipid compounds, and differentiating between live and dead organisms are among the notable difficulties [38]. *Brukina*, being a traditional fermented milk‐millet smoothie that is produced using artisanal processes, may contain a diverse range of microorganisms that may come from the environment, handling conditions, raw materials, and even vending conditions [2, 21]. Sampling such a heterogeneous microbial community can be challenging. The difficulty in obtaining representative samples, especially sampling at different fermentation stages or from diverse production environments, may limit the accuracy of microbial profiling [104, 138]. Variability in microbial community may also exist in different batches or packages of *Brukina* [138]. These factors should be accounted for in analyzing and interpreting metagenomics data from *Brukina.*

#### 2.12.2. Underrepresented Reference Database

Metagenomics relies heavily on the availability of comprehensive and accurate reference databases for taxonomic assignment and functional annotation [139]. Regarding traditional foods like *Brukina*, the functional annotation and reference genomes for the particular bacteria that control the fermentation process are limited [140]. This is because *Brukina* rely on complex microbial consortia originating from spontaneous fermentation and are underrepresented in the commonly used databases, SILVA and Greengenes. These databases are skewed toward model organisms or bacteria frequently seen in industrial food production, which makes it difficult to describe the microbial diversity of *Brukina′s* understudied microbes completely [139]. As a result, its metagenomic analyses suffer from incomplete taxonomic resolution or misclassification of novel taxa. Consequently, understanding the entire range of microbial activity during fermentation is severely limited by the poor taxonomic classification and functional profiling caused by this underrepresentation [132, 139, 140].

#### 2.12.3. Lack of Standardization of Methodologies

The differences in metagenomic methods, such as the choice of DNA sequencing approaches (targeted sequencing and WGS) and bioinformatic tools for computational analysis, often led to varying results [65, 141]. Accordingly, direct comparisons between studies are difficult. This is particularly because various methods have different advantages and disadvantages based on the varying algorithms on which these tools operate [141, 142]. Therefore, pipeline selection can have a big influence on how data are interpreted. For instance, shotgun metagenomics examines the complete microbial community, whereas 16S rRNA amplicon sequencing concentrates on a single marker gene [142, 143]. Additionally, disparities in taxonomic designation and functional predictions may result from the use of various reference databases and bioinformatics methods [144]. Standardized procedures for sample collection, DNA extraction, sequencing depth, and data processing are lacking in the *Brukina* case [2]. This discrepancy lowers the dependability and comparability of results by making it challenging to create reproducible workflows. Additionally, the resolution of microbial diversity and the identification of functional genes might be impacted by the sequencing technology selection, such as between Illumina and Oxford Nanopore [66].

#### 2.12.4. Food Safety Concerns

Naturally occurring bacteria are involved in the fermentation of *Brukina*, and if the fermentation process is not closely monitored, some of these microbes may have the potential to be harmful [3, 21, 145]. Ensuring food safety in *Brukina* requires the identification and monitoring of foodborne pathogens, spoilage organisms, and bacteria resistant to antibiotics [3, 21]. Nevertheless, metagenomic methods are sometimes constrained by their inability to identify spoilage organisms or low‐abundance diseases unless sequencing depths are high enough, which can be expensive and time consuming [26, 146]. Furthermore, insufficient reference databases and erroneous functional gene annotations may result in false positives or incorrect identification of potentially dangerous microorganisms [147].

### 2.13. Future Directions

#### 2.13.1. Enhancing Reference Database

One of the key future directions in *Brukina* metagenomics is the improvement of reference databases for microbial taxonomy and functional gene annotation. Expanding these databases to include indigenous microorganisms found in traditional fermented foods like *Brukina* will enhance the accuracy and completeness of taxonomic classifications and functional gene predictions. This could involve efforts to sequence the genomes of microorganisms commonly associated with West African fermented foods, enriching the reference libraries, and allowing for more precise identification and functional analysis of fermentation‐related microorganisms.

#### 2.13.2. Development of Standardized Methodologies

Future research should also concentrate on creating uniform procedures for sample collection, DNA extraction, sequencing, and data processing to address the lack of standardization in metagenomic studies. This will make it possible to compare the microbial diversity of several batches of *Brukina* and other fermented foods more effectively and guarantee consistency and repeatability between studies. Standardized data reporting formats will also make it easier for researchers looking into traditional fermented foods to collaborate and share data.

#### 2.13.3. Integration of Multi‐Omics Approach

To further understand the fermentation process, future studies could investigate the combination of metagenomics with other omics approaches, such as proteomics and metabolomics. The dynamic interactions between microorganisms, substrates, and fermentation products can be better understood using multi‐omics techniques. Metabolomic profiles and metagenomic data, for instance, may be combined to help discover important metabolic processes in *Brukina* that produce tastes and bioactive chemicals. A comprehensive understanding of the fermentation process and its effects on food safety and quality would be possible with such integrated techniques.

#### 2.13.4. Public Health and Policy Implications

It will be crucial to take into account the public health and regulatory ramifications as metagenomic research on *Brukina* expands. Future research ought to examine how metagenomic information can influence laws governing the safety of traditional fermented foods and the conditions under which they are produced and vended. Additionally, preventing contamination and promoting the safe consumption of *Brukina* would be made possible by making sure that regional food safety procedures are in line with scientific developments in microbial ecology and fermentation.

## 3. Conclusion

*Brukina* is more than just a fermented milk and millet smoothie; it is a culturally significant, nutrient‐dense beverage deeply rooted in West African tradition. However, its artisanal production process presents both opportunities and challenges from a microbiological perspective. Although spontaneous fermentation improves digestibility, flavor, and certain health advantages, it also adds unpredictability and potential proliferation of opportunistic microorganisms.

The application of metagenomics provides a revolutionary perspective for investigating the microbial ecology and functional potential of *Brukina.* This is exemplified by NGS technologies such as targeted amplicon sequencing and shotgun sequencing, which have been employed to characterize entire microbial communities, uncover biochemical pathways, and identify functional genes relevant to fermentation processes and quality attributes in various fermented foods. Nonetheless, there are still issues with sample complexity, reference database restrictions, inconsistent methodology, and food safety. Enhancing microbial databases, standardizing procedures, incorporating multi‐omics methodologies, and boosting pathogen detection skills should be the main goals of future research. Therefore, there is a critical need for specialized metagenomic research on *Brukina*, which is still poorly understood in comparison to other fermented foods, as highlighted by this literature review. By tackling these issues, metagenomic techniques can be extremely helpful in maximizing *Brukina* fermentation, guaranteeing food safety, and maintaining the customs that give this product its distinctive character.

## Funding

No funding was received for this manuscript.

## Conflicts of Interest

The authors declare no conflicts of interest.

## Data Availability

Data sharing not applicable to this article as no datasets were generated or analyzed during the current study.
